# The Role of Exercises in Osteoporotic Fracture Prevention and Current Care Gaps. Where Are We Now? Recent Updates

**DOI:** 10.5041/RMMJ.10308

**Published:** 2017-07-31

**Authors:** Helen Senderovich, Henry Tang, Samuel Belmont

**Affiliations:** 1 Geriatrics & Palliative Care & Pain Medicine, Baycrest Health Sciences, Assistant Professor, University of Toronto, Department of Family and Community Medicine, Division of Palliative Care, Toronto, Canada; 2 Baycrest Health Sciences, Toronto, Canada

**Keywords:** Bone mineral density, elderly, exercise, fracture, osteoporosis

## Abstract

**Introduction:**

The primary non-pharmacological management recommended for patients with osteoporosis (OP) is exercise, but whether it should be high-force, resistive, or other means can be obscure.

**Objective:**

To describe the role of exercises in osteoporotic fracture prevention, identify effects and potential risks of high-force exercises, detect the optimal exercises to combat OP, and explore the challenges that might arise from interventions.

**Methods:**

A search on MEDLINE and Cochrane databases was conducted on the role of exercises in preventing osteoporotic fractures from 1989 onwards, leading to 40 results, including op-ed pieces, qualitative studies, randomized clinical trials (RCTs) (*n*=5), and RCT follow-up studies (*n*=1). Articles deemed relevant to the objectives were analyzed and summarized. Data on effects of vitamin D and calcium supplementation were later gathered from different sources as well.

**Results:**

High-intensity, resistive strength training provided the maximum benefit in increasing bone mineral density (BMD) levels, muscle mass, and reduction in fractures, while posture and balance exercises only improved mobility. High-force exercises did not increase fractures and were associated with increases in BMD. Interventions including exercises, vitamin D, and calcium intake had limited effect when used as single interventions, while vitamin D and calcium may potentially cause increases of cardiovascular events.

**Conclusion:**

A long-term regular exercise program designed to improve postural stability, mobility, and mechanical efficiency, alongside an increased vitamin D and dietary calcium intake, is most effective in preventing OP and reducing osteoporotic fractures.

## INTRODUCTION

Osteoporosis (OP) is an incurable weakening of bone structure due to loss of tissue from mineral deficiency in the elderly. It is the most common type of bone disease,^1^ and the low bone mineral density (BMD) leads to an increased risk of skeletal fractures, most often in the hip, wrist, or vertebrae. Osteoporotic fractures are one of the leading causes of death in the elderly.^2^ Current treatment and management are limited and focused on regular screening and pharmacological interventions. However, non-pharmacological options including exercises cannot be overlooked and can be just as critical in maintaining bone health, although experts disagree on the most effective forms of exercise. High-force training is effective in developing bone and muscle mass, but may increase risk of trauma,^3^ while low-force training is safer but potentially lacks in terms of BMD growth. In this review, we concentrated on the role and difference in impact of high- and low-force exercises on osteoporotic fractures. Vigorous, short, and repetitive doses of mechanical loads were found to reduce hip fracture risk, and increase BMD.^4^ Resistance and balance training were associated with better mobility and may reduce falls and subsequently prevent fractures.^5^ Moderate-force exercise, involving resistance and balance training, is recommended to the elderly and may reduce risk and fear of fractures, although its effectiveness is conditional.

Recent estimates state over 200 million people have OP,^6^ a number that is expected to increase in the following years. By 2050, hip fracture cases are estimated to increase by 310% and 240% in men and women, respectively, compared to rates in 1990.^7^ The greatest consequence of OP is an increased risk and fear of fractures: over 8.9 million fractures, which stem from falls to small stresses, are caused annually by this disease.^7^

Unfortunately, OP produces no symptoms before fractures, making detection difficult. As of now there is no treatment, and the increasing prevalence of fractures makes diagnosis and the management of the disease all the more critical. Risk factors that may contribute to the development of OP include aging, weight lower than 60 kg, family heredity, medication such as steroids, smoking, and becoming postmenopausal for women. However, regular screening and injury are the only ways accurately to diagnose the disease, both of which are tedious. By that point, OP could have progressed to a dangerous level, and the damage might have already become extraordinarily severe: disabilities caused by OP exceed those caused by all cancers except lung cancer.^7^ Although certain tests, such as a timed “up and go,” have been shown to be a sensitive and accurate measure for recurring fractures,^8^ there have not been many experiments to definitely validate the procedure’s effectiveness.

There is currently no treatment for OP, although pharmacological drugs such as bisphosphonate, estrogens, and parathyroid hormone (teriparatide) have been shown to be able to slow down or pause the breakdown of bone tissue, although not without side effects.^9^ The non-pharmacological option of exercises is more often recommended as it can be more effective than drugs and is widely accessible. The best method of exercise, however, remains a debatable topic. Maintaining or increasing mobility, muscle strength, BMD, bone mineral content (BMC), and balance requires regular, moderate to vigorous, high-force exercise. It is also possible that such force and high-impact exercises may result in more falls and have similar effectiveness in increasing BMD as low-force and low-intensity training,^3^ although evidence in this area appears to be lacking. The exact repertoire of exercise varies from person to person; nevertheless, this review will explore the general qualities required for all fracture-reducing exercises.

Our objective was to describe the effects and potential risks of high-force exercise, identify the optimal exercises to combat OP, and explore the challenges that might arise from the intervention.

## METHOD

We conducted a MEDLINE as well as a Cochrane databases search on recent literature regarding the role of exercises in prevention of osteoporotic fractures to get this review started. We limited our search to the years from 1989 onwards. This yielded about 40 results ranging from op-ed pieces to qualitative studies, to randomized clinical trials (RCTs) (*n*=5) and RCT follow-up studies (*n*=1), to prospective cohort studies (*n*=6), as well as cross-sectional studies (*n*=2) and non-randomized controlled clinical trials (*n*=1). We identified relevant systematic reviews and meta-analyses (*n*=7) from these databases and checked their reference lists, as well as the RCTs included in the review, to supplement the search. Of these, only approximately 16 were deemed to be useful and relevant to the objective at hand. These articles were then read and the relevant points and themes were summarized. Corroborative themes were identified, and the authors responsible for the contributing research were cited as they came up. Additional sources were added to the literature search after scrutinizing the papers’ reference lists. Trials in which participants were not at risk of OP were excluded, as well as trials in which only high-trauma fractures were recorded as an outcome.

A summary of articles reviewed is found in Table 1.

**Table 1:** Summary of Articles from MEDLINE and Cochrane Databases Search.

Author (Year) Ref. #	Study Design	Population	Instruments and Outcomes	Results and Author’s Conclusions
Howe et al. (2011)^10^	Systematic review 43 RCTs; 4,320 participants met inclusion criteria	Healthy postmenopausal women (including those with previous fractures) aged 45–70 years	Number of incident fractures: vertebral and non-vertebral (hip and wrist) BMD, BMC, or calcium bone index immediately post-intervention and at follow-up BMD measured by single-photon absorptiometry, dual-photon absorptiometry, quantitative CT or DXA at baseline, immediately post-intervention, and at follow-up Serious adverse events including death Minor adverse events including falls	Most effective exercise intervention on BMD for neck or femur appears to be non-weight-bearing high-force exercise Most effective intervention for BMD at the spine was combination exercise compared with control groups For postmenopausal women: ○ Exercise slightly improves BMD ○ Exercise slightly reduces chances of fracture
Ebrahim et al. (1997)^11^	RCT	Caucasian postmenopausal women 165 women with history of upper limb fracture in past 2 years; selected from local accident reports and emergency departments	Spinal fractures BMD at the femoral neck Lumbar spine BMD	No significant differences in clinical or spinal X-ray fracture risk or self-rated health status Promotion of brisk walking exercise by nursing staff may have a small, but clinically important, impact on BMD, but is associated with increased risk of falls
Preisinger et al. (1996)^12^	RCT	92 sedentary postmenopausal women aged 45–75 years with low back pain for at least 1 year and sedentary lifestyle for past 10 years	BMD and bone width calculated through the mean of four distal (distal forearm) and six proximal (mid-forearm) scans distal to a radio-ulnar distance of 8 mm Occurrence of bone fractures recorded by standardized questionnaire	Long-term regular exercise program designed to improve postural stability, mobility, motor control, coordination, and mechanical efficiency is effective in preventing OP and may delay bone loss
Sinaki et al. (2002)^13^	10-year follow-up study to a RCT	Postmenopausal women, aged 58–75 years	BMD, spine radiographs, back extensor strength, biochemical marker values, and level of physical activity obtained for all subjects at baseline, 2 years, and 10 years	Significant difference in BMD at 10-year follow-up Long-term effect of strong back muscles in reducing vertebral fractures in estrogen-deficient women
Nelson et al. (1994)^14^	RCT	40 sedentary and estrogen-deplete postmenopausal white women, aged 50–70 years	Femoral neck and lumbar spine BMD DXA for bone status, one repetition maximum for muscle strength, 24-hour urinary creatinine for muscle mass, and backward tandem walk for dynamic balance	Femoral neck BMD, lumbar spine BMD, total body BMC, muscle mass, muscle strength, and dynamic balance: Increased in strength-trained women; Decreased in controls High-intensity strength training exercises are an effective means to preserve BMD while improving muscle mass, strength, and balance in postmenopausal women Maintaining spinal BMD and improving strength and balance can significantly reduce the risk of falls and fractures
Uusi-Rasi et al. (1999)^15^	Cross-sectional study	117 healthy, female postmenopausal recreational gymnasts (mean age 62.1 [SD 4.7] years) and 116 sedentary controls (mean age 61.5 [4.6] years)	BMC of the distal radius, femoral neck and trochanter, measured with DXA BMC of the mid-shaft and distal tibia and trabecular density of the distal tibia, measured with peripheral quantitative CT	Recreational gymnastics was significantly associated with higher BMC and trabecular density at the tibia only Recreational gymnastics and folk dancing improve muscular strength and increase bone mass and bone size in the tibia Osteoporotic fractures are uncommon in the tibia; long-term recreational gymnastics is especially beneficial for muscular performance and body balance, which may translate to decreased propensity to fractures among elderly people
Rikkonen et al. (2010)^16^	15-year follow-up to population-based OP risk factor and prevention (OSTPRE) study	8,560 postmenopausal women; mean age 52.2 years at baseline	Areas of BMD at the proximal femur and lumbar spine, followed at 5-year intervals with DXA Data concerning physical activity, fractures, and falls, registered at 5-year intervals with postal enquiries	Physical activity is associated with a moderate rise in wrist fracture risk but does not increase risk of other fractures, and might significantly decrease proximal femur bone loss among postmenopausal women
Kemmler et al. (2015)^17^	Non-RCT 16-year follow-up analysis	105 postmenopausal osteopenic women	Clinical overall low-trauma fractures determined by questionnaires, structured interviews, and BMD at the lumbar spine and femoral neck, assessed by DXA	Clear evidence of high anti-fracture efficiency of multipurpose exercise programs
Kemmler et al. (2013)^18^	Systematic review and meta-analysis	Subjects 45 years and older	“Overall fractures” (i.e. cumulated number of fractures) and vertebral fractures assessed by radiographs of spine	Evidence that exercise reduces overall and, to a lesser degree, vertebral fractures in the elderly
Moayyeri (2008)^3^	Meta-analysis of 13 prospective cohort studies	Many different-aged cohorts, all participants at least 40, male and female	Meta-analysis of 13 prospective cohort studies with hip fracture end-point presented	Moderate-to-vigorous physical activity is associated with reduced risk of hip fracture Risk of falling generally reduced among physically active people; potential increased risk in the most active/inactive people Positive effects of physical activity on BMD are of questionable magnitude for reducing fracture risk
Lock et al. (2006)^19^	Systematic review and meta-analysis of RCTs	Caucasian postmenopausal women	Spinal fractures (all trials), wrist fractures, and fractures at any site (one trial) X-rays of the thoracic and lumbar spine and one of the lateral spine (two trials)	In all trials, exercise associated with a non-significantly lower risk of spinal fractures
Yoshimura (2003)^20^	Literature review		Literature search over past 13 years to assess the relationship between exercise and the risk for low bone mass and OP-related fractures	One RCT showed back-stretching exercise reduced the risk for vertebral fractures High-impact and/or weight-bearing exercise might increase bone density in the elderly
Gregg et al. (2000)^21^	Review and synthesis of published literature		MEDLINE literature search including RCTs, case-control, and prospective cohort studies on the effects of physical activity on the incidence of falls and fracture risk	Higher levels of leisure time physical activity prevent hip fractures, and certain exercise programs may reduce risk of falls

BMD, bone mineral density; BMC, bone mineral content; CT, computerized tomography; DXA, dual-energy X-ray absorptiometry; OP, osteoporosis; RCT, randomized control trial.

## DISCUSSION

### Effects of High-Force Exercise

The immediate step to take for any individual desiring to strengthen bone health is to undergo regular vigorous exercise. Through extensive study, high-intensity exercise has been examined and found to produce several positive effects. Analysis has indicated that exercises may serve to maintain mobility, physicality, BMD, muscle, strength, and balance.^22^ One RCT found that high-intensity strength training, when employed twice a week, proved to be a feasible means to preserve bone density while improving muscle mass, strength, and balance in postmenopausal women.^14^ Together, these benefits attributed to a small but statistically significant reduction in falls, particularly when the exercise program involved balance and lower-body strength training, which may be associated with a reduction of up to 40% in hip fractures.^21^ In a meta-analysis of 13 cohort studies, with all participants over the age of 40, moderate to vigorous exercise was found to reduce hip fractures by 45% and 38% in men and women, respectively.^3^ The potential benefits of a low-intensity program were less pronounced, as they may initially fail to prevent bone loss and lead to increased fall risk, despite increased muscle strength.^11^,^23^ However, when examined at a 10-year long-term follow-up, there was evidence that a low-intensity program can indeed lead to a reduction in fractures.^13^

Despite concern from many osteoporotic patients that exercise increases the risk of fractures, data suggested that injury rates actually decline,^1^ and severe damage was non-existent. Several experiments in the past decade involving the elderly (mean age around 70) undergoing several months of exercise programs that featured balance, resistance, and walking showed improvements in maximum standing time, which may indicate lower risk of falls and subsequent fractures. Serious detrimental effects including new vertebral fractures or wavering cardiovascular systems were not found.^24^ The intensity could be considered moderate to intense, although no significant high-force exercise was practiced. For residents at high risk of fractures, balance, strength, and functional training exercises were suggested only as a part of a multifactorial intervention to prevent falls. Importantly, this recommendation was primarily focused upon avoiding the small increase in falls which may occur among high-risk individuals when participating in exercises.^25^

It should be noted that identifying patients at high risk of fracture does not necessarily require BMD testing.^26^ Patients who have had prior hip fracture or vertebral fracture, recently used systemic glucocorticoids and have had one prior fracture, or who have been identified as at high risk due to repeated falls, and/or are on OP treatment are all considered at high risk of fracture.^26^

Another benefit exercise had on patients with OP was a reduced fear of falling. A RCT involving 89 elderly women who undertook a 3-month exercise program revealed that patients with a history of osteoporotic fractures had significantly lowered risk of falling and associated fractures, and diminished fear of falling. Alongside the exercise training, the patients also engaged in educational sessions on fall prevention strategies. Together, these interventions were found to have a durable effect, possibly because patients felt confident upon learning that exercise does not usually increase fractures.^27^

Despite the noted benefits, those who decide to engage in exercise must choose to be cautious as it is highly conditional. Although moderate or vigorous exercise was shown to have a positive relationship with muscle and peak BMD,^28^ those who are extremely inactive should not take part in such intense activities initially, because their behavior might contribute to a potentially increased risk of falls when first exercising and subsequently result in fractures.^3^ A low-to-moderate-intensity workout on a regular schedule was found to be the best way to deal with such situations.^4^ One study had also associated exercise with a mild increase in wrist fractures;^29^ the exact reason for this is unknown. It had not been found to increase fractures in other areas of the body.

There was not enough solid evidence about high-force exercises to strongly conclude whether they are detrimental or beneficial. However, they were not found to result in more fractures than normal^20^,^21^ and were noted to be able to increase BMD in young women, although this might be in part due to the small population samples. The elderly, who have more frail immune systems, lower strength, and weaker regenerative capabilities compared to the younger population, would not be advised to participate in high-force activities.^30^ However, high impact did not translate to high intensity, which in fact greatly improved BMD and muscle strength with minimal injury.^28^ In addition, moderate-force activities were found to reduce risk of fractures.^31^ Swimming, strength training, or even walking for extended distances was found to be effective in significantly decreasing urinary deoxypyridinoline which is related to a reduction in bone resorption.^32^ The chance of falls, which may correlate to the chance of fractures, was practically non-existent, while the intensity was moderate to high.^32^

### Types of Exercise

There was great variation in optimal exercise strategy between people, which can stem from ethnicities, background, education, health care, wealth, nutrition, and genetics. However, research indicated that certain groups of exercise were more effective at counterbalancing the effects of OP. Fractures and BMD vary by exercise type. Osteoporosis Canada lists four different essential forms of exercise: strength training, posture training, balance training, and aerobic training.

Strength training, also known as resistance training, was the most effective.^33^ The primary objective of strength training is to increase muscle mass, which is often an issue with the elderly; by age 70 people are expected to lose between 50% and 55% of their maximum muscle mass. The low muscle mass can allow for easy falls resulting in fractures that might seem impossible for another person in the same situation. Strength training may also increase BMD in certain parts of the body, including the spine and hip bone,^4^,^34^ as was evident in one RCT of strength-trained postmenopausal women.^14^ As OP is a common complication that results in hip fracture following a fall, increasing BMD in the hip may prevent further fractures^35^ and reduce the risk of fractures being related to OP. Improvements in spinal strength is also critical as OP is often associated with spinal disability and deformity. A RCT involving a 10-year follow-up found the incidence of vertebral compression fracture in postmenopausal women who underwent progressive, resistive, back-strengthening exercises for 2 years was only 1.6% compared to the control group, which had an incidence rate of 4.3%.^13^ However, contrary to that study, a systematic review and meta-analysis of RCTs found that, during all trials, exercise only resulted in a non-significantly lower risk of spinal fractures.^19^ Nevertheless, the most effective type of exercise intervention on BMD for the neck of the femur is non-weight-bearing high-force exercise such as progressive resistance strength training for the lower limbs.^10^ Walking was also included in this category, as it made the patient work against the force of gravity. Data pointed to high levels of walking being able to reduce bone resorption in elderly women and maintain levels of quantitative ultrasound parameters.^32^ As walking is relatively low risk, it is highly recommended although it cannot replace other strength training exercises.

Posture training involves proper alignment and positioning of various body parts, especially the spine. Kyphosis, or an arching of the back, naturally begins to develop as people age. However, weak extensor muscles along the back can result in excessive kyphosis, which is often the cause of fractures. Although OP fractures in the spine rarely cause paralysis as fractures occur in the lower or middle area of the spine, they can cause disabilities, gradual loss of mobility, and death. The aim of posture training is to strengthen muscles along the back and the hip, the areas most affected by OP, and improve alignment of the spine so small twists and loads upon the vertebrae will not result in fractures.^5^,^36^ This is especially important for patients with spine fractures in order to prevent repeated fractures. Posture training includes exercises that stretch the back and stomach area. None of these exercises are high-force, as even small to moderate amounts of increased load on the back may result in fractures.

Balance training exercises are those that work to improve balance and reduce falls. A total of 20%–30% of Canadians fall each year,^37^ and, as falls are the most common trigger for fractures for patients with OP, enhancing balance and coordination is critical in reducing fractures.^38^ These exercises are mostly aimed at enhancing leg muscles which allows patients to be steadier when standing up or moving. Results demonstrated greater mobility and improved functional and static balance. Long-term balance training reduced fear of falling and increased isometric strength and standing time.^24^,^39^ Balance training can be practiced anywhere.

In a RCT, 65 healthy Caucasian women were tested to study the effect of non-loading exercise on bone aging. During the 2-year study, bone loss did not vary statistically from the intervention group to the control group,^23^ meaning posture and balance exercises did not increase BMD and might be ineffective in reducing fracture risk in the short term. However, muscle mass increased noticeably.^23^ Long-term effects are different; a follow-up conducted to this study found that the difference in bone mineral density, while not significant between the two groups at baseline and 2-year follow-up, was significant at 10-year follow-up.^13^

Aerobic training is any physical activity that increases heart and breathing rate, and can be performed continuously for several minutes at a time. The purpose of aerobic training is to strengthen the heart and lungs, as well as other muscles within the body, and improve endurance. This specific type of exercise overlaps with the previous three. Weight-bearing and resistance aerobic exercise is recommended for patients with OP, as it manages to increase both muscle mass and bone density, reducing the chance of fractures.^5^ Recreational gymnastics or folk dancing are also considered aerobic activities, and while they increase muscular performance they are associated with higher BMD only at the tibia.^15^

According to Canada’s Physical Activity Guide, all adults are encouraged to undergo at least 30 minutes of moderate to vigorous aerobic exercises 5 days a week, even for elderly with OP and previous fractures.^5^ An extra hour of exercise a week for women in moderate-intensity sporting translates to a 0.8% increase in hip BMD,^40^ and a 7%–8% increase in peak BMD results in a 50% reduction in fracture risk.^28^ Although women are four times as likely to develop OP due to their smaller bone mass and longer life span compared to men, exercise should still be performed by the male population.

As there may be no single optimal exercise strategy, a multimodal exercise program incorporating many of the strategies above should be considered, as long-term follow-ups of multipurpose exercise programs usually evidence clinical low-trauma fractures and general anti-fracture efficiency.^17^ One RCT combining high-velocity progressive resistance training, weight-bearing impact, and challenging balance or mobility activities resulted in significant net gains in femoral neck and lumbar spine BMD. It posits that a multimodal exercise program represents an effective approach to improving multiple musculoskeletal and functional performance measures in older, at-risk adults.^29^ Another study found exercise involving a combination of balance and lower-extremity strength training may reduce risk of falling.^21^ Further large-scale trials are needed to evaluate the efficacy of this multimodal approach on reducing falls and fractures.^18^

### Potential Barriers

Those who have no former habit of exercise cannot hope to improve significantly upon their osteoporotic condition. High-stress and weight-bearing exercises had been shown to have a positive correlation with increase in peak bone mass, which reduces fracture risk.^41^ After menopause, however, research showed that bone mass density will start to decrease even under the influence of exercise.^42^ Seniors with OP would be unable to make significant progress in increasing BMD, and can only reduce or prevent deterioration. Muscle mass begins to decline earlier. It is unlikely the body would remain competent at old age, even under the influence of exercise. Adults are advised to exercise regularly at moderate to vigorous intensities well into old age, to ensure that they can effectively combat or prevent the disease from developing or progressing.

Another widely noted barrier is the lack of symptoms for patients with OP. The only signs OP showed before a painful fracture was stooped posture, loss of height and back pain, all of which could be attributed to natural aging of the body and degenerative disc disease. Thus, OP was often undetected until a fracture occurred, by which time mobility might have become limited and recovery near impossible. Even amongst long-term care (LTC) patients, there is a 40% mortality rate within a year following a hip fracture.^43^ For the remaining residents, although a certain extent of mobility could be recovered, only 40% of patients were able to return to their former living standard after the fracture,^7^ and at least 10% re-fractured within a year.^43^ Once again, the most successful single intervention is regular exercise, which will delay or prevent the progression of OP and prevent fractures from ever occurring in the first place.^12^ Occasional quantitative ultrasound screening is also recommended.^32^

Interestingly, one RCT concluded that only undergoing the single intervention of exercise produces results far from optimal and may have negative results.^44^ It did note, however, that large-scale efforts are necessary to bring meaning to the findings. While exercises alone being insufficient had been previously documented, more trials must be carried out to validate the potential negative effects of exercise.

### Vitamin D and Calcium Supplementation

While exercises are considered one of the most important methods of OP management, there are other available interventions. In particular, calcium and vitamin D supplements are often recommended to patients, as these two substances are vital in maintaining bone health and are lacking in patients with OP. Calcium builds and maintains bones, while vitamin D protects bones and allows the body to absorb stored calcium.^45^ Osteoporosis Canada recommends daily vitamin D supplements of 800 to 2,000 IU for adults over 50 who are at high risk of fractures, as well as those with OP or vitamin D deficiency; younger adults need at least 400 IU of vitamin D intake from supplements every day.^46^ Intakes of over 2,000 IU for any age can produce a number of side effects including skin reactions, sleepiness, nausea, vomiting, and changes to blood sugar and blood pressure.^47^ Vitamin D deficiency or excess also impacts cardiovascular death risk and falls.^48^ Vitamin D 25 OH Levels should be around 70 nm/L in blood and remain in the range of 50–100 nm/L to achieve the benefit.^49^ As for calcium, which the body does not naturally produce, adults over 50 are recommended by OP Canada to have dietary supplementation equal to three servings of dairy products daily which is equivalent to 1,200 mg of elemental Ca, while adults under 50 should take two servings. For residents at high risk who cannot meet the dietary calcium requirement, supplements containing up to 500 mg of elemental calcium daily is recommended.^50^ Like with vitamin D, there must be strict regulations as excess calcium can cause kidney stones, fatigue, depression, and hypercalcemia.^51^

However, similar to exercise, vitamin D supplementation has been shown to be ineffective in reducing falls and to have a limited effect, especially when used as a single intervention.^52^ While the Scientific Advisory Council of OP Canada recommend daily supplements for those at high risk of fracture, other sources claim it should not be taken without other forms of intervention.^52^ For example, vitamin D taken alongside calcium probably reduces hip fractures and mortality more than vitamin D alone or calcium alone. For residents at high risk, such an intervention results in an estimated 15/ 1,000 fewer hip fractures.^53^–^55^

Calcium supplementation is also ineffective when used as the primary defense against OP and fractures, and has been found to provide minor improvements to BMD, even when taken alongside vitamin D.^56^ In addition, calcium supplementation and cardiovascular risk seemingly have a positive relationship. Calcium supplementation is believed to contribute to the development of cardiovascular events such as myocardial infarction, and stroke in rare scenarios, but there is limited evidence suggesting a correlation.^56^ Some studies, such as a NIH-AARP Diet and Health prospective study consisting of several RCTs from 2013, discovered an increased risk of cardiovascular events from calcium supplementation, which they reasoned likely arose from vascular calcification caused by the artificial intake.^57^ Most research, such as a 2015 meta-analysis of postmenopausal women, found no relationship.^58^

Extensive evidence shows that dietary calcium, the calcium received from natural sources of food, has an inverse relationship with cardiovascular disease and other blood disorders. A meta-analysis from 2016 found a 9% decrease in stroke risk for every 200 g of total fermented dairy intake per day.^59^ As a result, greater dietary calcium intake is encouraged over calcium supplements.

Besides being an important mineral for bone health, however, calcium aids with muscle contraction and is in constant use when exercising.^45^ To compensate for the drop in calcium levels as the body consumes it through physical activity, extra calcium from the bones is released, which can further lower BMD in patients with OP and effectively defeat the purpose of exercise.^60^ Further research should be devoted to determining whether exercises are best done alongside taking calcium and vitamin D supplements, and the amount that is considered tolerable, and how it relates to the well-known guidelines and existing controversies. Evidence shows that exercises increase BMD, but there could potentially be a threshold when it is instead detrimental. Only low-quality evidence is available to show that multifactorial interventions reduce risk of hip fractures (10 per 1,000 fewer: 95% CI).^61^

Determining such a threshold is a critical step in ensuring that patients can optimally benefit from this intervention.

### Patient Management Recommendations

Traditional management of patients with osteoporosis revolves around pharmacological treatments as well as screening. There have been few studies on pharmacological drugs in the prevention of recurring fractures, but apparently the evidence is strong enough to justify their use even though there are potential side effects.^62^ However, many patients with previous fractures fail to receive the proper drugs. An investigation from Belgium identified 23,146 patients who had previously sustained hip fractures and were hospitalized. Out of them, only 6% received anti-osteoporotic pharmacological treatment.^63^ Thus, a priority is to ensure all patients receive the proper interventions to suit their needs.

Interventions such as exercise, vitamin D, and calcium supplementation are disregarded to an even greater extent.^64^ Firstly, and most importantly, the patients must have a regular exercise program with emphasis on resistance or strength training, which has maximum benefit and a low risk of falls or fracture.^10^,^33^ Staff must continuously encourage patients to undergo the exercise, as many patients might not participate willingly. Patients should also practice balance training exercises such as Tai Chi, a Chinese martial art, which was associated with a 47.5% reduction in fall risk during a RCT.^65^ In addition to enhancing mobility, balance training also decreases the risk of falling and improves patients’ self-perceived health.^5^ It does not, however, reduce the risk of fracture should a patient fall, only the occurrence of falls.^62^

Patients who have had vertebral compression fractures or have abnormal back structures should also be encouraged to wear thoracolumbar braces, a type of brace that attempts to straighten the spine. Rigid thoracolumbar braces forcibly hold up the spine with the goal to develop proper back structures. However, some patients dislike the lack of mobility of these rigid braces, and instead will use softer braces, such as a posture-training support brace.^64^ The purpose of such braces, which are essentially weights, is to constantly remind patients to straighten their mid-backs. In a pilot study, braces were found to reduce back pain among patients with acute vertebral compression fracture, with 17 out of 23 patients reporting significant improvement.^66^ On the other hand, hip protectors, which are meant to reduce the impact of falls to the hip, are controversial. Long-term compliance is an unresolved issue, and many studies have not reported noticeable benefit.^62^

Patients should have regular intake of vitamin D and calcium. Since the body naturally synthesizes vitamin D upon exposure to UVB sunlight radiation,^67^ patients should be taken outside, with a caregiver or personal support worker (PSW), for 5–10 minutes of exposure at least twice a week near midday, when UVB radiation is strongest.^64^ Patients should also consider taking vitamin D supplementation, in the range of 800–2,000 IU.^46^ If temperatures are cold or there is no option for sunlight exposure, patients should consider simply taking the supplementation. However, this is just a guideline; the quantity and exact method must be evaluated on a case-by-case basis, and interventions should only be implemented with the informed consent of the patient.

Calcium supplementation should be around three servings of dairy products daily, and can be taken in multiple forms, such as calcium citrate and calcium carbonate.^50^ Calcium supplementation is preferably to be taken alongside meals, as many forms only function as desired with acids.^64^ Similar to vitamin D, the risks of calcium supplementation should be considered on individual basis. Generally, dietary calcium supplementation, as opposed to artificial calcium supplementation, is safer and preferred.

Other, equally important, simple but pro-active approaches exist that reduce the chance of falls. Notably, efforts should be made to reduce clutter and environmental distractions, which can affect the ease of navigation.^62^ In addition, use of devices that aid in movement, such as walkers, should also be considered depending on the patient’s condition.

Long-term care facilities, in particular, must seek to incorporate daily moderate-intensity exercise sessions for patients, with emphasis on posture, balance, and strength resistance exercises. However, there must be careful evaluation of which patients can partake in these exercise sessions, as the risk of adverse events such as falls is increased for those who are most inactive. The LTC facility should obtain information regarding the prior activity levels of candidate patients, in order to restrict the participation of patients in the exercise sessions as necessary. Ideally, strict inclusion/exclusion criteria should be developed, which would enable LTC employees or PSWs to determine which patients can be recommended to undergo regular exercise and what type of intervention would be individually suitable.

Potential barriers to the integration of said exercise programs include the cost of the necessary equipment, as well as the cost of running multiple tailored exercise programs. The potential staff training requirements must be considered, and the staff must remain vigilant in their monitoring of patient progress in order to ensure that no adverse effects are being experienced. Exercise specialists such as kinesiologists, personal trainers, and physiotherapists could be used to train non-specialized LTC hospital staff initially, so that the exercise programs may remain cost-effective. One extensive systematic review found that effective group-based exercise programs can be implemented in LTC hospitals with monetary resource constraints, with the use of trained staff members (e.g. nurses, PSWs, and volunteers), using simple and inexpensive equipment, and carried out three times per week for 30–45 minutes per session.^68^ More research should determine how to optimize the feasibility of exercise programs specifically targeted to the elderly population residing in different long-term settings and/or their homes, and a patient inclusion/exclusion guideline should be created.

### Areas of Future Research

Osteoporosis is a “silent disease” because its symptoms are almost unrecognizable and attacks without warning. This is in part due to the lack of symptoms but also due to the limited knowledge and information regarding its risk factors, which play just as big a role in identifying diseases. Further research is needed to identify and prevent OP before it develops, knowing that exercise can only provide a mild improvement to the condition.^11^ If we were to have an outline and a table for the risk factors of OP, we could identify high-risk elderly individuals and, by getting them to undergo exercise, possibly prevent OP from ever causing fractures.

Furthermore, while there is evidence that exercise reduces fractures to a certain extent, publication bias may have weakened the results. More RCTs and follow-ups regarding the relationship between exercises and fractures in all prone sections of the body, with vertebral fractures as a primary end-point, should be carried out.

## CONCLUSION

Despite previous belief that exercise contributes to fractures, it has clearly been identified as a viable method for combating BMD and muscle loss and reducing falls and fractures. All seniors, including those who have a history of OP and fractures, are highly recommended to undergo daily, medium-force training sessions with emphasis on posture, balance, and most importantly strength resistance exercises at a minimum of moderate intensity. Exercises should also be balanced into the work life of young adults to ensure BMD and muscle mass are still present into the senior years. The exact age to start is still unclear. Care and caution must be taken when exercising as injuries and fractures can still happen in the process. For this reason, high-force exercises should be considered with extreme caution: although they have been shown to have some significant positive impact, they should be avoided by all those who are inexperienced or have significantly limited strength and mobility; medium-force exercise is preferred. Exercises alone, however, are insufficient to halt the course of the disease and may have adverse side effects. The elderly are advised to undergo regular screening, as knowledge about risk factors is limited, in order for the disease to be diagnosed early, and to use multiple interventions possibly including vitamin D supplementation alongside exercise. It is uncertain how much recovery is possible after a fracture has occurred, but exercising in moderation is undoubtedly an essential step that must be taken.

## Abbreviations

BMC: bone mineral content
BMD: bone mineral density
OP: osteoporosis
PSW: personal support worker
RCT: randomized control trial.
